# Rapid Progression of Pulmonary Blastomycosis in an Untreated Patient of Chronic Lymphocytic Leukemia

**DOI:** 10.1155/2014/514382

**Published:** 2014-04-13

**Authors:** Pralay K. Sarkar, Paras Malhotra, P. S. Sriram

**Affiliations:** ^1^Divisions of Pulmonary, Critical Care and Sleep Medicine, Department of Medicine, University of Florida, Gainesville, FL 32608, USA; ^2^Pulmonary and Critical Care Medicine, Baylor College of Medicine, One Baylor Plaza, Houston, TX 77030, USA; ^3^Ben Taub General Hospital, 1504 Taub Loop, BTGH-6PF80002, Houston, TX 77030, USA

## Abstract

Chronic lymphocytic leukemia (CLL) is associated with a state of immunosuppression characterized by hypogammaglobulinemia as well as B and T lymphocyte dysfunction. Though opportunistic infections are common in CLL patients, particularly after treatment, reports of infections by endemic dimorphic fungi are very few. Here we report a case of pulmonary blastomycosis in a CLL patient who initially presented with an indolent pulmonary mass lesion. The pulmonary lesions progressed rapidly over a two-week period. The diagnosis was established by transbronchial lung biopsy. He was treated with Amphotericin B lipid complex followed by oral itraconazole and recovered uneventfully. This case illustrates the importance of a timely diagnosis and treatment. The presentation of blastomycosis in immunocompromised patients, diagnosis, and treatment are discussed.

## 1. Introduction

Blastomycosis refers to an endemic mycosis caused by the dimorphic fungus* Blastomyces dermatitidis*. It is endemic in midwestern, south-eastern, and south central United States. In immunocompromised patients, disseminated infection has been reported. Here we described a case of rapidly progressive pulmonary blastomycosis in a patient with CLL.

## 2. Case Report

**Figure 1:**
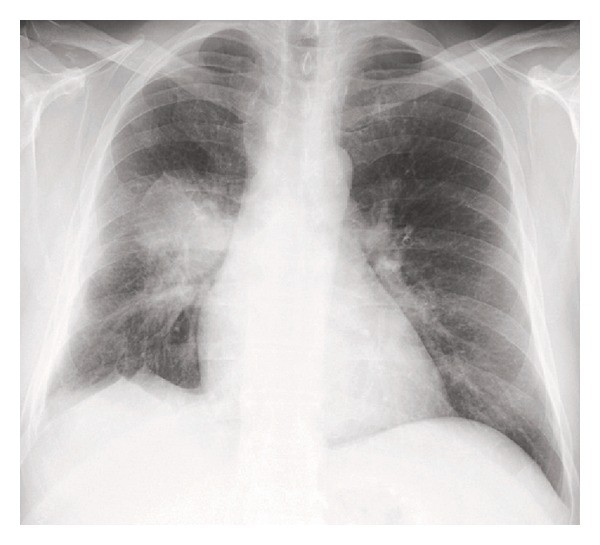
CXR showing right lower lobe mass lesion at the time of presentation.

**Figure 2:**
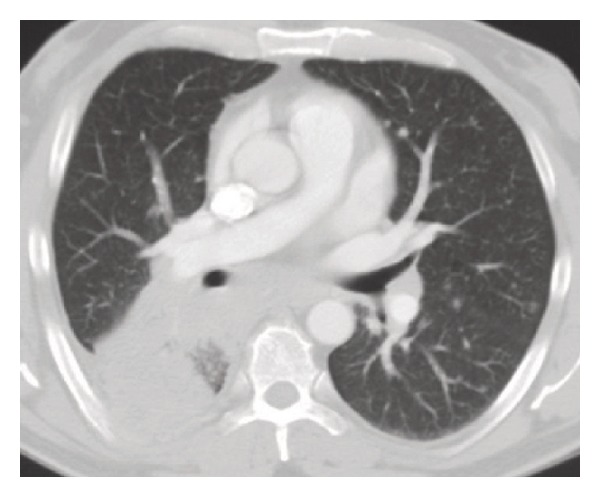
CT scan chest at presentation showing areas of consolidation in the right lower lobe. Also note the small right pleural effusion.

**Figure 3:**
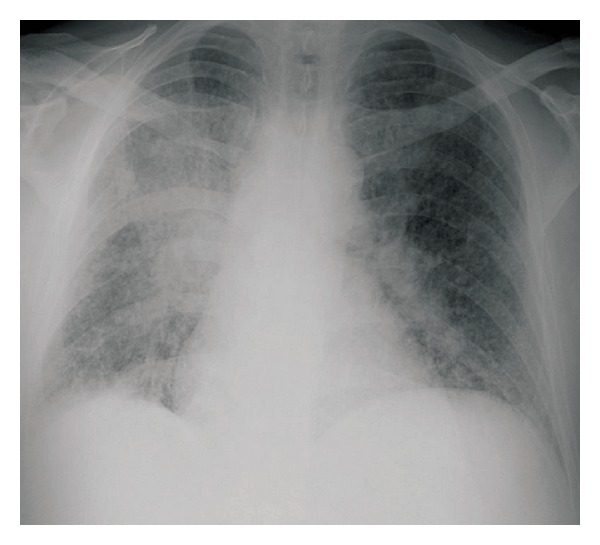
CXR showing progression of right lower lobe lesion and development of bilateral miliary nodules. There was 2-week time interval between this CXR and CXR shown in Figure 1.

**Figure 4:**
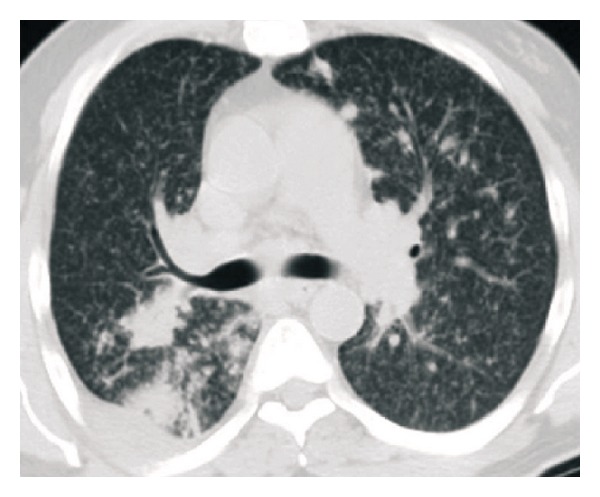
CT scan obtained 2 weeks following the initial evaluation confirmed the miliary dissemination, progression of the index mass lesion, and several new satellite lesions.

**Figure 5:**
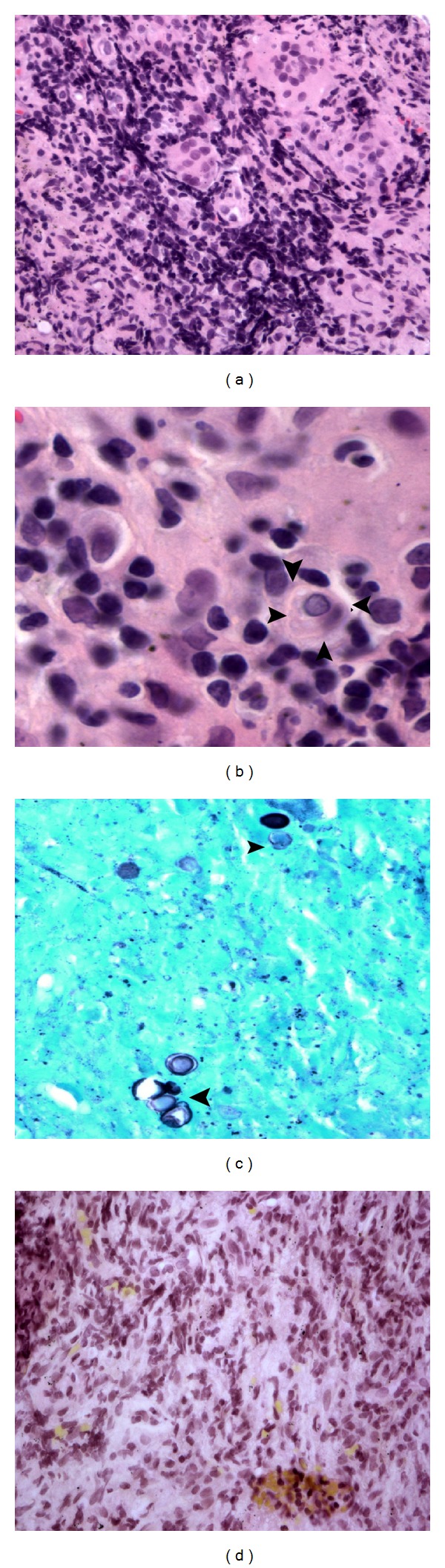
Representative photomicrographs from the transbronchial lung biopsy. (a) Lymphocytic infiltration and multiple giant cells (H & E, ×200). (b) Intracellular fungal organism within a giant cell (arrowheads) (H & E stain ×600). (c) Yeast-like cells of* Blastomyces dermatitidis*. Note the characteristic broad based budding (arrowhead) (GMS stain, ×400). (d) A representative section with negative mucicarmine stain (×200).

A 50-year-old nonsmoker man was referred to the pulmonary clinic with 4-week history of nonproductive cough, right sided pleuritic chest pain, night sweats, and unquantified weight loss. He did not have any hemoptysis, dyspnea, or wheezing. He was diagnosed to have B-cell chronic lymphocytic leukemia (B-CLL) 4 years prior to this presentation. However, as he was asymptomatic, no treatment was required for CLL till this presentation. His other medical history included a diagnosis of type 2 diabetes mellitus. His vital signs at the time of our initial evaluation were as follows: temperature 37°C, heart rate 75 beats/min, blood pressure 105/63 mm Hg, respiratory rate 18/min, and oxygen saturation 98% on room air. On physical examination, he had no evident respiratory distress. He had generalized lymphadenopathy, consistent with his known diagnosis of CLL. Examination of the respiratory system revealed bilateral vesicular breath sounds without any adventitious sounds. Examination of cardiovascular system, abdomen, and central nervous system was unremarkable. Laboratory examination revealed a white blood cell count of 117,000/mm^3^ (1% neutrophils, 96% lymphocytes, and 2% monocytes), 1.4% hemoglobin 8.9 g/dL, hematocrit 28.1%, and platelet count 243,000/mm^3^. Serum electrolytes, renal function, and liver function tests were normal. Chest X-ray (CXR) revealed a right lower lobe mass (Figure 1). CT scan chest revealed right lower lobe mass with mediastinal invasion and small right pleural effusion (Figure 2). A possible diagnosis of primary bronchogenic carcinoma was entertained and the patient underwent an outpatient fiberoptic bronchoscopy. Bronchoscopy did not reveal any endobronchial lesion. Bronchoalveolar lavage (BAL) and a transbronchial lung biopsy were obtained from right lower lobe segments. Cytological analysis of BAL showed a mixed inflammatory infiltrate with predominant lymphocytes. One week after the bronchoscopy, before the biopsy report was available, he was admitted in view of marked fatigue and weakness. CXR showed progression of the radiological changes, in the intervening two weeks from the initial imaging, with a new development of a miliary pattern (Figure 3). CT scan obtained at this time confirmed the miliary dissemination (Figure 4). The patient did not report any worsening of his respiratory symptoms or any extrapulmonary symptoms. The transbronchial lung biopsy results from the right lower lobe returned and showed numerous lymphocytes, histiocytes, and many multinucleated giant cells, forming ill-defined granulomas (Figure 5(a)). The biopsy also revealed presence of thick-walled single-yeast forms of approximately 8–15 *μ*m size (Figure 5(b)). The organisms stained positively with Gömöri methenamine silver (GMS) stain and did not stain with mucicarmine (Figures 5(c) and 5(d)). Some of these yeast forms showed eccentric broad based budding (Figure 5(c)), overall morphology consistent with blastomycosis. Bronchial biopsy and BAL cultures did not show any growth of fungus in 4 weeks. CT scan of abdomen and pelvis did not show any lesion in liver, spleen, or kidneys. Magnetic resonance imaging of the brain did not show any lesion. Cerebrospinal fluid analysis was normal. The patient received eight days of treatment with Amphotericin B lipid complex at a dose of 5 mg/kg body weight. Treatment was subsequently continued with itraconazole 400 mg/day for 12 months. Follow-up imaging of the lungs showed satisfactory resolution of the pulmonary lesions. The patient was continued indefinitely on itraconazole 200 mg/day as he subsequently required chemotherapy for management of CLL.

## 3. Discussion

In this report, we have a described a case of rapid progression of pulmonary blastomycosis, from a single mass lesion to miliary dissemination over 2 weeks, in a patient with untreated CLL. This case highlights the importance of prompt diagnosis and institution of treatment in immunocompromised patients. Though our patient did not develop respiratory failure in spite of miliary involvement of the lungs, blastomycosis is known to cause acute respiratory distress syndrome with a high mortality [1] and therefore an early diagnosis leads to a favorable outcome. Blastomycosis has been reported in association with different immunosuppressive states, including a treated case of CLL with resultant absolute neutropenia [2]; our patient had rapid progression to disseminated disease in absence of neutropenia or recent chemotherapy. Our patient did not have any history of living in or traveling to a zone known to be endemic for blastomycosis. This case highlights the difficulty of diagnosing a sporadic case of blastomycosis in nonendemic areas.

CLL is characterized by a state of immunodeficiency and infection is a common cause of death [3]. Along with the well-known abnormality of hypogammaglobulinemia, there is also evidence of defects in cell mediated immunity, relevant in causing predisposition to intracellular pathogens like* Blastomyces* spp. In most patients with CLL, there is an increase in both CD8+ and CD4+ T-cell numbers and reversal of CD4/CD8 ratio; however, it has been suggested that the T cell in B-CLL may be unable to start, maintain, and complete an immune response to the malignant B cell and other antigens, the result being susceptibility to infections and sustenance of the tumor [4]. Defects in phagocytic function and cytotoxic activity of neutrophils, monocytes, and natural killer (NK) cells and defects in complement system are other immunological changes described in CLL [5]. As a defect in cell-mediated immunity will predict, CLL patients are susceptible to a host of opportunistic infections, for example,* Listeria* spp.,* Nocardia* spp.,* Candida* spp.,* Aspergillus* spp.,* Pneumocystis jirovecii*,* Histoplasma capsulatum*,* Cryptococcus neoformans* and atypical mycobacteria; these do not occur commonly in untreated patients. As patients are treated, depending on the type of treatment agent (alkylating agent, purine analogues, or monoclonal antibodies), somewhat different clinical spectrums of opportunistic infections are seen [6].

A wide variety of radiographic changes have been reported in pulmonary blastomycosis; radiological findings have been reported to be largely similar in immunocompromised and immunocompetent patients [7, 8]. Common chest radiological patterns in blastomycosis include alveolar infiltrates, solitary pulmonary nodules, lung mass with or without cavitation, interstitial infiltrates, mediastinal lymphadenopathy, and pleural involvement [8]. Alveolar infiltrates are more common in acute presentation, whereas mass like lesions are more common in chronic presentation. CT chest similarly shows a broad range of findings: mass lesions, consolidation with air bronchogram, intermediate-sized nodules, satellite lesions around mass or consolidation, pleural thickening, small effusions, and cavitation [9]. There is no pathognomonic radiological pattern in blastomycosis [10, 11]; therefore, presentation in a nonendemic area with a mass-like lesion may raise obvious diagnostic difficulties, as happened in our index case. Miliary pattern is less commonly seen. We speculate that the rapid progression of the pulmonary lesions in our case, from a solitary lesion to a miliary pattern, resulted from erosion into a pulmonary vessel; however, an earlier autopsy report of a case of miliary blastomycosis did not identify vascular invasion [12]. Systemic dissemination is likely in a case of miliary dissemination; however, our patient did not have any evidence of clinical disease in any extrapulmonary site.

Diagnosis can be established by direct visualization of the fungus in clinical specimen or culture of body fluid or tissue. Rapid diagnosis can be established by microscopic examination of sputum or bronchial washing, digested with 10% potassium hydroxide.* B. dermatitidis* can be identified by its characteristic morphology: the yeast-like cells of the fungus are oval to spherical, 8–15 *μ*m in diameter, and with thick refractile wall. They are multinucleate and show single broad-based budding: useful diagnostic features. Different stains can be used to demonstrate the organism. Papanicolaou staining of respiratory tract specimens has a high diagnostic yield (93%); the organism appears to be refractile and pale blue-green with this stain. Other useful special stains are Gömöri methenamine-silver (GMS) or periodic acid-Schiff (PAS) stains. Identification of* B. dermatitidis* in hematoxylin-eosin (H & E) stained histopathologic specimens is often difficult. Mucicarmine stain is useful in differentiation from capsule deficient forms of* Cryptococcus neoformans*, being negative in case of* B. dermatitidis*. The respiratory tract specimens were sterile in our index case; however, microorganisms have been recovered in a high percentage of cases with documented pulmonary disease [13].

Amphotericin B (AmB) remains the drug of choice in blastomycosis with life threatening disease or with CNS involvement, for patients who are immunocompromised or for whom azole treatment has failed [14, 15]. For moderate to severe pulmonary disease, current guidelines recommend treatment with a lipid formulation of AmB at a dosage of 3–5 mg/kg per day or AmB deoxycholate at a dosage of 0.7–1 mg/kg per day for 1-2 weeks or until improvement is noted, followed by oral itraconazole [15]. However, the cost and toxicity are substantial. Among the azole antifungals, ketoconazole was the first one used with success in treatment of blastomycosis. However, in the current guidelines, itraconazole has replaced ketoconazole as the azole of choice for its better antimycotic effect, better tolerability, and higher (up to 95%) rate of cure [15]. Treatment duration is 6–12 months or till resolution of radiological abnormalities.

There are no standard guidelines for antimicrobial prophylaxis in CLL patients. Clinical practice therefore varies, and in most cases, the prophylaxis is directed against herpes viruses,* Pneumocystis jirovecii,* and toxoplasmosis (e.g., acyclovir, sulfamethoxazole, and trimethoprim). A good case of antifungal prophylaxis is in selected clinical scenarios: treatment with either purine analogue or alemtuzumab, following stem cell allografts, previous systemic fungal infection, severe and prolonged neutropenia, and use of high-dose steroids and several previous rounds of immunosuppressive therapy [3].

## 4. Conclusion

CLL is characterized by a state of immunosuppression even in untreated patients. Blastomycosis is an endemic fungal infection and, particularly in an immunocompromised patient, can progress rapidly. Timely diagnosis helps in successful outcome. Amphotericin B and itraconazole are currently recommended treatments.

## References

[1] Lemos LB, Baliga M, Guo M (2001). Acute respiratory distress syndrome and blastomycosis: presentation of nine cases and review of the literature. *Annals of Diagnostic Pathology*.

[2] Isotalo PA, Ford JC, Veinot JP (2002). Miliary blastomycosis developing in an immunocompromised host with chronic lymphocytic leukaemia. *Pathology*.

[3] Hamblin AD, Hamblin TJ (2008). The immunodeficiency of chronic lymphocytic leukaemia. *British Medical Bulletin*.

[4] Scrivener S, Goddard RV, Kaminski ER, Prentice AG (2003). Abnormal T-cell function in B-cell chronic lymphocytic leukaemia. *Leukemia and Lymphoma*.

[5] Ravandi F, O’Brien S (2006). Immune defects in patients with chronic lymphocytic leukemia. *Cancer Immunology, Immunotherapy*.

[6] Morrison VA (2010). Infectious complications of chronic lymphocytic leukaemia: pathogenesis, spectrum of infection, preventive approaches. *Best Practice and Research: Clinical Haematology*.

[7] Recht LD, Davies SF, Eckman MR, Sarosi GA (1982). Blastomycosis in immunosuppressed patients. *American Review of Respiratory Disease*.

[8] Patel RG, Patel B, Petrini MF, Carter RR, Griffith J (1999). Clinical presentation, radiographic findings, and diagnostic methods of pulmonary blastomycosis: a review of 100 consecutive cases. *Southern Medical Journal*.

[9] Winer-Muram HT, Beals DH, Cole FH (1992). Blastomycosis of the lung: CT features. *Radiology*.

[10] Brown LR, Swensen SJ, Van Scoy RE, Prakash UBS, Coles DT, Colby TV (1991). Roentgenologic features of pulmonary blastomycosis. *Mayo Clinic Proceedings*.

[11] Sheflin JR, Campbell JA, Thompson GP (1990). Pulmonary blastomycosis: findings on chest radiographs in 63 patients. *The American Journal of Roentgenology*.

[12] Kar PM, Montalvo FA, Salazar DM, Kar SM (2009). Blastomycosis: case report investigating a persistent pulmonary lesion in an immunocompromised patient. *The Journal of the Kentucky Medical Association*.

[13] Martynowicz MA, Prakash UBS (2002). Pulmonary blastomycosis: an appraisal of diagnostic techniques. *Chest*.

[14] Chapman SW, Bradsher R.W. J, Campbell G.D. J, Pappas PG, Kauffman CA (2000). Practice guidelines for the management of patients with blastomycosis. *Clinical Infectious Diseases*.

[15] Chapman SW, Dismukes WE, Proia LA (2008). Clinical practice guidelines for the management of blastomycosis: 2008 Update by the infectious diseases society of America. *Clinical Infectious Diseases*.

